# Degradation Products of Polychlorinated Biphenyls and Their In Vitro Transformation by Ligninolytic Fungi

**DOI:** 10.3390/toxics9040081

**Published:** 2021-04-08

**Authors:** Kamila Šrédlová, Kateřina Šírová, Tatiana Stella, Tomáš Cajthaml

**Affiliations:** 1 Faculty of Science, Institute for Environmental Studies, Charles University, Benátská 2, 12801 Prague 2, Czech Republic; kamila.sredlova@biomed.cas.cz (K.Š.); katerina.sirova@biomed.cas.cz (K.Š.); 2 Institute of Microbiology of the Czech Academy of Sciences, Vídeňská 1083, 14220 Prague 4, Czech Republic; tatiana.stella@m3r.it; 3 M3R S.r.l., University of Milano Bicocca, Piazza della Scienza 1, 20126 Milano, Italy

**Keywords:** hydroxylated PCBs, chlorobenzyl alcohols, chlorobenzaldehydes, biodegradation, *Pleurotus ostreatus*, *Irpex lacteus*

## Abstract

Metabolites of polychlorinated biphenyls (PCBs)—hydroxylated PCBs (OH-PCBs), chlorobenzyl alcohols (CB-OHs), and chlorobenzaldehydes (CB-CHOs)—were incubated in vitro with the extracellular liquid of *Pleurotus ostreatus*, which contains mainly laccase and low manganese-dependent peroxidase (MnP) activity. The enzymes were able to decrease the amount of most of the tested OH-PCBs by > 80% within 1 h; the removal of more recalcitrant OH-PCBs was greatly enhanced by the addition of the laccase mediator syringaldehyde. Conversely, glutathione substantially hindered the reaction, suggesting that it acted as a laccase inhibitor. Hydroxylated dibenzofuran and chlorobenzoic acid were identified as transformation products of OH-PCBs. The extracellular enzymes also oxidized the CB-OHs to the corresponding CB-CHOs on the order of hours to days; however, the mediated and nonmediated setups exhibited only slight differences, and the participating enzymes could not be determined. When CB-CHOs were used as the substrates, only partial transformation was observed. In an additional experiment, the extracellular liquid of *Irpex lacteus*, which contains predominantly MnP, was able to efficiently transform CB-CHOs with the aid of glutathione; mono- and di-chloroacetophenones were detected as transformation products. These results demonstrate that extracellular enzymes of ligninolytic fungi can act on a wide range of PCB metabolites, emphasizing their potential for bioremediation.

## 1. Introduction

Ligninolytic (white-rot) fungi produce extracellular enzymes, such as lignin peroxidase (LiP; EC 1.11.1.14), manganese-dependent peroxidase (MnP; EC 1.11.1.13), versatile peroxidase (EC 1.11.1.16), and laccase (an oxidase; EC 1.10.3.2), which are pivotal for the natural decomposition of wood because they can break down the complex polymers of lignin. Because of the low substrate specificity of extracellular enzymes, ligninolytic fungi have also been extensively studied for their capacity to break down numerous environmental pollutants, including polychlorinated biphenyls (PCBs)—recalcitrant anthropogenic compounds with well-known adverse effects [1,2]. Ligninolytic fungi are also able to degrade or transform some of the degradation products of PCBs, namely, hydroxylated PCBs (OH-PCBs), chlorobenzoic acids (CBAs), chlorobenzyl alcohols (CB-OHs), and chlorobenzaldehydes (CB-CHOs) [1,2,3,4].

OH-PCBs are considered environmental pollutants and have been detected in plant, animal and human tissues, surface water, precipitation, and sediments [5,6,7,8,9,10,11]. The main source of OH-PCBs seems to be hydroxylation of legacy PCBs by organisms in the environment; nevertheless, some amount can also be formed abiotically, e.g., by reaction with ubiquitous hydroxyl radicals [12]. Recently, OH-PCBs were detected in sewage sludge, which can also constitute their source [13]. Aerobic bacteria can transform lower-chlorinated PCBs into their dihydroxylated metabolites by enzymes of the upper biphenyl pathway, while eukaryotic organisms usually employ cytochromes P450 to initially form monohydroxylated derivatives [14]. Despite the presence of the additional hydroxyl group, OH-PCBs are lipophilic compounds with partition coefficients that are comparable to their parent PCB congeners [15]. OH-PCBs have been identified as endocrine disruptors with various modes of action. For instance, due to structural similarities with thyroxine, they can bind to the transport protein transthyretin [16]. OH-PCBs can exhibit higher toxicity than the parent PCBs. In this regard, Kamata et al. [17] found higher affinities for the aryl hydrocarbon receptor of 26 out of 49 tested OH-PCBs when compared to those of the corresponding PCBs.

The transformation of OH-PCBs by ligninolytic fungi is catalysed by laccase; some isomers have been found to be oxidized only by a laccase –mediator system [3]. Schultz et al. [18] were the first to describe the transformation of OH-PCBs by laccase of the ligninolytic fungus *Pycnoporus cinnabarinus*. Depending on the position of the substituents, dimers were formed either with or without simultaneous dechlorination. Dimer formation was reported even with pentachlorinated OH-PCBs with laccase isoforms from *Trametes versicolor* [19]. In addition, Kordon et al. [20] detected dihydroxylated and quinone transformation products with laccases from *P. cinnabarinus* and *Myceliophthora thermophila*.

Unlike OH-PCBs, the first step in the transformation of CBAs by ligninolytic fungi is not provided by extracellular enzymes but by fungal cytochrome P450, resulting in the formation of a monohydroxylated metabolite [21]. Analogously to PCBs, during in vivo degradation of CBAs and OH-PCBs, the identified products included CB-OHs and CB-CHOs [2,4]. The study by Kamei et al. [2] is the only one where strains of ligninolytic fungi (*Phanerochaete* spp.) were incubated with these individual metabolites in vivo. However, very limited information is currently available on CB-OHs and CB-CHOs, and no in vitro studies exist that have addressed the structural limitations of their conversions.

The aim of this work was to assess the capabilities of the extracellular enzymes of *Pleurotus ostreatus* to biotransform selected PCB metabolites with additional analysis of the reaction products. *P. ostreatus* 3004 was selected as a representative of ligninolytic fungi, because this strain had been previously identified as an effective degrader of PCBs capable of lowering the concentration of even penta- and hexa-chlorinated congeners and suppressing acute toxicity during the process [1,22]. Extracellular enzymes exuded by this fungus in liquid medium were concentrated and used for in vitro biotransformation experiments with OH-PCBs, CB-CHOs, and CB-OHs with metabolite detection by GC–MS. Additional experiments were also performed with the extracellular enzymes of another ligninolytic fungus, *Irpex lacteus*. The employment of various mono- to tri-chlorinated compounds and the addition of enzyme mediators enabled us to assess the capability of the enzymes and susceptibility of different isomers for the transformation.

## 2. Materials and Methods

### 2.1. Materials and Chemicals

*P. ostreatus* 3004 CCBAS 278 and *I. lacteus* 617/93 were obtained from the Culture Collection of Basidiomycetes of the Czech Academy of Sciences.

Standards of 4-hydroxy-3,5-dichlorobiphenyl (4-OH-3,5-PCB; 100.0%); 4-hydroxy-2,2′,5′-trichlorobiphenyl (4-OH-2,2′,5′-PCB; 99.8%); and 4-hydroxy-2′,4′,6′-trichlorobiphenyl (4-OH-2′,4′,6′-PCB; 100.0%) were obtained from AccuStandard (New Haven, CT, USA). Standards of 2-hydroxy-5-chlorobiphenyl (2-OH-5-PCB); 4-hydroxy-2-chlorobiphenyl (4-OH-2-PCB); 4-hydroxy-3-chlorobiphenyl (4-OH-3-PCB); 4-hydroxy-4′-chlorobiphenyl (4-OH-4′-PCB); 2-hydroxy-3,5-dichlorobiphenyl (2-OH-3,5-PCB); 3-hydroxy-2′,5′-dichlorobiphenyl (3-OH-2′,5′-PCB); and 4-hydroxy-2′,5′-dichlorobiphenyl (4-OH-2′,5′-PCB) were kindly provided by Assoc. Prof. Ondřej Uhlík (University of Chemistry and Technology, Prague, Czech Republic).

Standards of 2-chlorobenzyl alcohol (2-CB-OH; 99%); 3-chlorobenzyl alcohol (3-CB-OH; 98%); 2,3-dichlorobenzyl alcohol (2,3-CB-OH; 97%); 2,6-dichlorobenzyl alcohol (2,6-CB-OH; 99%); 3,4-dichlorobenzyl alcohol (3,4-CB-OH; ≥98%); and 2,4,6-trichlorobenzyl alcohol (2,4,6-CB-OH; 97%) were purchased from Merck (Darmstadt, Germany). Standards of 4-chlorobenzyl alcohol (4-CB-OH; ≥99%); 2,4-dichlorobenzyl alcohol (2,4-CB-OH; ≥99%); 2,5-dichlorobenzyl alcohol (2,5-CB-OH; ≥99%); and 3,5-dichlorobenzyl alcohol (3,5-CB-OH; ≥98%) were purchased from Alfa Aesar (Ward Hill, MA, USA).

Standards of 2-chlorobenzaldehyde (2-CB-CHO; 99%); 3-chlorobenzaldehyde (3-CB-CHO; 97%); 4-chlorobenzaldehyde (4-CB-CHO; 97%); 2,3-dichlorobenzaldehyde (2,3-CB-CHO; 99%); 2,4-dichlorobenzaldehyde (2,4-CB-CHO; 99%); 2,5-dichlorobenzaldehyde (2,5-CB-CHO; 96%); 2,6-dichlorobenzaldehyde (2,6-CB-CHO; 99%); 3,4-dichlorobenzaldehyde (3,4-CB-CHO; 95%); 3,5-dichlorobenzaldehyde (3,5-CB-CHO; 97%); and 2,3,6-trichlorobenzaldehyde (2,3,6-CB-CHO; 97%) were purchased from Merck.

Syringaldehyde (SA; 98%); 1-hydroxybenzotriazole hydrate (HBT; 98%); 2-chlorobenzoic acid (98%), 3-chlorobenzoic acid (≥99%), 4-chlorobenzoic acid (PESTANAL^®^), glucose oxidase from *Aspergillus niger* (type X-S); dimethyl sulfoxide (DMSO; ≥99.5%); *N*,*N*-dimethylformamide (≥99.9%); L-glutathione reduced (≥98.0%); and 1-iodopropane (99%) were obtained from Merck. Ethylacetate (EtOAc; ≥99.8%), and acetone (≥99.8%) were purchased from VWR (Stříbrná Skalice, Czech Republic). All the other chemicals were of analytical grade or higher.

### 2.2. Extracellular Liquid Harvest

Malt-extract–glucose medium (MEG) contained 10 g L^−1^ of glucose and 5 g L^−1^ of malt extract broth (Oxoid, Basingstoke, UK). *P. ostreatus* culture was maintained at 4 °C on agar plates with MEG and 2% agar–agar Kobe I (Carl-Roth, Karlsruhe, Germany). The inoculum was prepared by placing three agar plugs of the culture (d = 1 cm) into 20 mL of MEG in 250-mL Erlenmeyer flasks. After cultivation (static, 7 days, 28 °C), the inoculum was homogenized by Ultra-Turrax T 25 (IKA, Staufen im Breisgau, Germany), and 5 mL of the homogenate was added to 45 mL of MEG in 500-mL Erlenmeyer flasks. After an additional 7 days of cultivation, the extracellular liquid was separated from the mycelium by a nylon mesh. The liquid was then gradually filtered through a series of glass fibre filters (1.4 and 0.5 µm; Macherey-Nagel, Düren, Germany) and cellulose nitrate membrane filters (0.45 and 0.2 µm; Whatman, GE Healthcare, Chicago, IL, USA) and concentrated approximately 250-fold using ultrafiltration discs (regenerated cellulose, molecular weight cut-off 10 kDa; Merck). The concentrated extracellular enzymes were stored at −20 °C and filtered through a 0.22-µm nylon syringe filter (Membrane Solutions, Auburn, WA, USA) before immediate use.

### 2.3. Enzyme Activity

The activities of laccase, MnP, manganese-independent peroxidase (MIP), and LiP in the extracellular liquids of *P. ostreatus* and *I. lacteus* and during the in vitro experiments were determined spectrophotometrically (Infinite M200 PRO microplate reader; Tecan, Männedorf, Switzerland). The in vitro samples in which enzyme activity was determined were set up as biotic controls (see Section 2.4.) and contained no mediators. Laccase was determined with 2,2′-azino-bis(3-ethylbenzothiazoline-6-sulfonic acid) (ABTS; ≥98%; Merck) as the substrate at 420 nm [23]. MnP and MIP activities were assessed from the oxidative coupling of 3-methyl-2-benzothiazolinone hydrazone (98%; Acros Organics, Geel, Belgium) and 3-(dimethylamino)benzoic acid (98%; Alfa Aesar) at 595 nm [24,25]. LiP was assessed with 3,4-dimethoxybenzyl alcohol (96%, Merck) as the substrate at 310 nm [26]. One enzyme unit (U) was defined as the amount of enzyme that converts 1 μmol of its respective substrate per minute.

### 2.4. In Vitro Biotransformation Experiment with Pleurotus ostreatus

The in vitro experiment was performed in glass vials; the total volume of the reaction mixture was 0.5 mL. All mixtures contained the extracellular liquid (for enzyme activities, see Section 3.1), 2 µg mL^−1^ of each of the 10 compounds from their respective groups (OH-PCBs, CB-OHs, or CB-CHOs), and 4% DMSO. The setup to favour laccase activity was carried out in 0.1 M sodium acetate buffer (pH 4.5) with either 1 mM SA, 1 mM HBT, or with no mediator. The setup favouring MnP activity was performed in 0.5 M sodium malonate buffer (pH 4.5) and contained 30 mM glucose with 0.06 U mL^−1^ of glucose oxidase (source of H_2_O_2_), 10 mM MnSO_4_, and either 5 mM glutathione or no mediator [27]. All sample setups were conducted in triplicate. Biotic controls were prepared analogously but contained no analytes. Heat-deactivated controls contained the same enzymes denatured by heat (1 h, 100 °C). Abiotic (buffer) controls were prepared with distilled water instead of the extracellular liquid. The samples were incubated on an orbital shaker (160 rpm, OS 5 basic, IKA) at 28 °C for 0, 1, 6, 24, 72, and 168 h. The reactions were stopped by the addition of 0.34 M NaCl, 1 mM H_2_SO_4_, and 2 mL of EtOAc.

### 2.5. In Vitro Biotransformation Experiment with Irpex lacteus and Chlorobenzaldehydes

An additional experiment was performed with the extracellular liquid of *I. lacteus* to assess whether a higher activity of MnP can transform CB-CHOs (*P. ostreatus* produces mainly laccase). The cultivation of *I. lacteus* and the concentration of the extracellular liquid were performed according to Linhartová et al. [28]. The reaction mixture (0.5 mL) contained the extracellular liquid (for enzyme activities, see Section 3.1), 2 µg mL^−1^ of each CB-CHO, 0.5 M sodium malonate buffer (pH 4.5), 30 mM glucose with 0.06 U mL^−1^ glucose oxidase (source of H_2_O_2_), 10 mM MnSO_4_, and either 5 mM glutathione or no mediator (conditions identical to the MnP-favouring setup with *P. ostreatus* in Section 2.4). The samples were incubated at 28 °C for 1, 24, and 72 h.

### 2.6. Sample Extraction and Derivatization

All samples were extracted 5x with 2 mL of EtOAc on a reciprocal shaker (20 min; LT3; Nedform, Valašské Meziříčí, Czech Republic) and dried using anhydrous sodium sulphate. Aliquots of the extracts were taken for derivatization and analyte determination by GC–MS. CB-CHOs were analysed without derivatization. OH-PCBs were propylated by 1-iodopropane: the aliquot was evaporated to dryness under a stream of N_2_ and reconstituted in 150 µL of acetone, after which approximately 30 mg of anhydrous K_2_CO_3_ and 50 µL of 1-iodopropane were added [29]. After derivatization (100 °C, 30 min), the sample was evaporated to dryness and reconstituted in EtOAc. CB-OHs were trimethylsilylated: the aliquots were added with 100 µL of *N*,*N*-dimethylformamide and evaporated to 100 µL. After the addition of 200 µL of a mixture of *N*,*O*-bis(trimethylsilyl)trifluoroacetamide and trimethylchlorosilane (99:1; Merck), the samples were derivatized for 30 min at 70 °C. The derivatization reagent was removed by evaporation under N_2_ before GC–MS analysis.

### 2.7. GC–MS Analysis

The analytes were determined using the SCION SQ (Bruker, Billerica, MA, USA) equipped with the CP8400 autosampler (Agilent Technologies, Santa Clara, CA, USA) and a DB-5MS column (30 m, 0.25 mm ID, 0.25 µm; Agilent Technologies). The basic conditions were identical for all analysed compounds. The sample (1 µL) was injected at 240 °C, the split ratio was 1:50, and the column flow was 1.4 mL min^−1^ (99.999% helium; Linde, Prague, Czech Republic). The transfer line and ion source temperatures were 280 and 250 °C, respectively.

The GC oven programme for OH-PCBs was as follows: 60 °C (1 min hold), 60–160 °C at 30 °C min^−1^, and 160–240 °C at 4 °C min^−1^ (10 min hold). Two monochlorinated OH-PCBs (4-OH-2-PCB and 4-OH-3-PCB) shared the same retention time under the given conditions; therefore, the data were processed and presented as a single compound. The GC oven programme for CB-OHs was as follows: 60 °C (1 min hold), 60–80 °C at 20 °C min^−1^, 80–140 °C at 3 °C min^−1^, and 80–240 °C at 25 °C min^−1^ (10 min hold). The GC oven programme for CB-CHOs was as follows: 40 °C (1 min hold), 40–100 °C at 8 °C min^−1^, 100–120 °C at 2 °C min^−1^, 120–130 °C at 5 °C min^−1^, and 130–240 °C at 25 °C min^−1^ (10 min hold).

The analytes were identified by comparison to their corresponding standards; selected ion monitoring was used for quantification. The chromatograms of the derivatized and nonderivatized samples were also inspected for the presence of metabolites. Additional metabolite identifications were performed from the total ion current by referencing with the NIST/EPA/NIH Mass Spectral Library (NIST 14; National Institute of Standards and Technology, Gaithersburg, MD, USA), available standard compounds, and/or by individual interpretation of mass fragmentations (electron ionization, 70 eV).

### 2.8. Data Analysis

Figures were plotted using the OriginPro 8.5 software (OriginLab Corporation, Northampton, MA, USA). Statistical analysis was processed in the software R (version 3.5.1; R Core Team, 2018, Vienna, Austria).

## 3. Results

### 3.1. Enzyme Activity

The concentrated extracellular liquid of *P. ostreatus* contained predominantly laccase and a lower activity of MnP. No MIP or LiP was detected. At the start of the experiment, the in vitro mixtures were set up to contain 450 ± 9 U L^−1^ of laccase, and the activity of MnP was 30 ± 2 U L^−1^. During the in vitro experiment, enzyme activity was measured in samples set up as biotic controls with no mediators to assess enzyme stability. The activity of laccase in the laccase-favouring setup steadily decreased to 131 ± 3 U L^−1^ after 168 h. The activity of MnP in the MnP-favouring setup was only 1.2 ± 0.2 U L^−1^ after 72 h and was not detected at 168 h.

The concentrated extracellular liquid of *I. lacteus* contained only MnP and MIP. No laccase or LiP was detected. The in vitro experiment was set up to contain 706 ± 4 U L^−1^ of MnP and 114 ± 1 U L^−1^ of MIP. At the end of the experiment (72 h), the activities of MnP and MIP were 290 ± 20 U L^-1^ and 85.6 ± 0.4 U L^−1^, respectively.

### 3.2. Biotransformation of Hydroxylated Polychlorinated Biphenyls

Different conditions were applied to aid the transformation capabilities of the two detected enzymes of *P. ostreatus*. The laccase setup was performed with or without the addition of mediators—SA (compound widely present in nature) or HBT (synthetic mediator). The MnP setup was supplemented by an H_2_O_2_-generating system, Mn^2+^ ions, and glutathione or no mediator.

**Figure 1:**
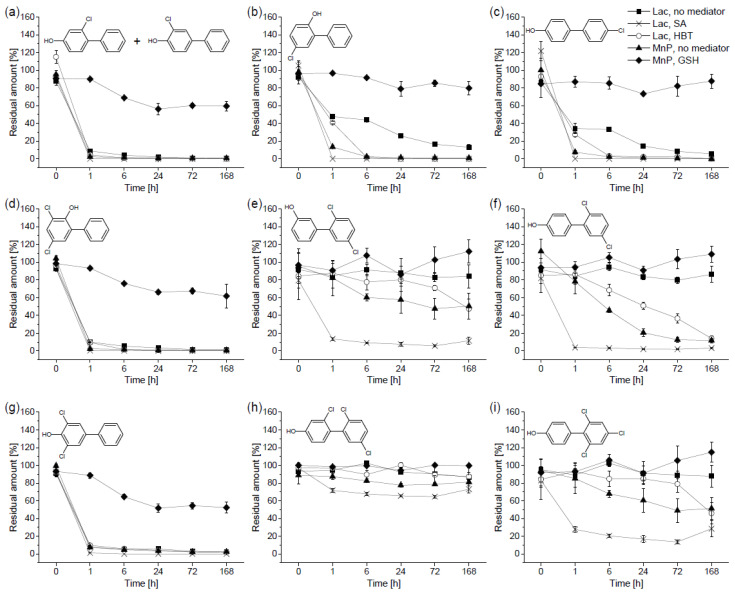
Residual amounts (related to corresponding heat-deactivated controls) of hydroxylated polychlorinated biphenyls (OH-PCBs) obtained during the in vitro experiment with the extracellular liquid of *Pleurotus ostreatus*: (**a**) 4-hydroxy-2-chlorobiphenyl and 4-hydroxy-3-chlorobiphenyl; (**b**) 2-hydroxy-5-chlorobiphenyl; (**c**) 4-hydroxy-4’-chlorobiphenyl; (**d**) 2-hydroxy-3,5-dichlorobiphenyl; (**e**) 3-hydroxy-2’,5’-dichlorobiphenyl; (**f**) 4-hydroxy-2’,5’-dichlorobiphenyl; (**g**) 4-hydroxy-3,5-dichlorobiphenyl; (**h**) 4-hydroxy-2,2’,5’-trichlorobiphenyl; and (**i**) 4-hydroxy-2’,4’,6’-trichlorobiphenyl. The OH-PCBs were incubated in a mixture; the initial concentration was 2 µg mL^−1^ for each. The initial enzyme activities were 450 U L^−1^ of laccase and 30 U L^−1^ of manganese-dependent peroxidase (MnP). The laccase-favouring setup (Lac) contained no mediator (◼), syringaldehyde (SA; ✕), or 1-hydroxybenzotriazole (HBT; ◯); the MnP-favouring setup contained no mediator (▲) or glutathione (GSH; ⯁).

As shown in Figure 1, the extracellular liquid of *P. ostreatus* was able to transform most of the tested OH-PCB congeners; the addition of HBT, and especially SA, greatly enhanced the removal of some of them. In general, the rate and extent of the removal negatively correlated with the level of chlorination and was dependent on the position of the chlorine substituents. In this regard, all monochlorinated congeners (4-OH-2-PCB + 4-OH-3-PCB, 2-OH-5-PCB, and 4-OH-4′-PCB; Figure 1a–c) as well as 2-OH-3,5-PCB (Figure 1d) and 4-OH-3,5-PCB (Figure 1g), which have both chlorine substituents and the hydroxyl group on the same aromatic ring, were completely or nearly completely removed within the 7 day incubation, most of them within the first hour, particularly in the presence of SA. Interestingly, the removal of 2-OH-3,5-PCB and 4-OH-3,5-PCB without a mediator was better than the removal of monochlorinated 2-OH-5-PCB and 4-OH-4′-PCB. In contrast, 3-OH-2′,5′-PCB (Figure 1e) and 4-OH-2′,5′-PCB (Figure 1f) were only transformed in the presence of mediators in the laccase setup; SA performed substantially better than HBT. Regarding the two tested trichlorinated OH-PCBs, 4-OH-2′,4′,6′-PCB (Figure 1i) was susceptible to transformation (residual amount of only approximately 30% with the aid of SA after 7 days), while no apparent depletion trend was observed for 4-OH-2,2′,5′-PCB (Figure 1h).

The results of the nonmediated reactions containing glucose, glucose oxidase, and MnSO_4_ (the MnP setup) were comparable for some congeners to the results of the laccase-favouring setups. Noticeably, the removal of 4-OH-2’,5’-PCB was even better in the nonmediated MnP setup than in the HBT-mediated laccase setup. Nevertheless, the addition of glutathione completely or nearly completely hindered the biotransformation of all OH-PCB congeners, suggesting that glutathione acted as a laccase inhibitor and confirming that the main enzyme involved in the transformation of OH-PCBs was indeed laccase.

In samples derivatized by 1-iodopropane, GC–MS analysis revealed a metabolite with an *m*/*z* of 156, which was present in trace amounts after 24 and 72 h of incubation in the laccase-favouring setup with no mediator, SA, and HBT and in the MnP setup with no mediator. The compound was identified according to the mass spectrum as monochlorinated CBA (Figure S1). The molecular ion (*m*/*z* 198 belonging to the propylated CBA) was missing from the spectrum; this was also the case with standards of the three possible isomers of monochlorinated CBAs, which were used for comparison of the mass fragmentation and retention times.

Another monochlorinated metabolite with an *m*/*z* of 260 was detected in samples derivatized by 1-iodopropane that was not present in nonderivatized samples (Figure S2). This compound was found in trace amounts in samples with active laccase (laccase setup with no mediator, SA, and HBT, and the MnP setup with no mediator) but in none of the controls (heat-deactivated, abiotic, or biotic). It was also absent in the MnP setup supplemented with glutathione in which laccase was inhibited. The compound was most abundant after 1 h of incubation; at 24 h and thereafter, it was not present. It exhibited an initial loss of −42 (propyl, indicating the presence of at least one hydroxyl group) and additional losses of −29, −63, and −92, which are typical for hydroxylated polychlorinated dibenzofurans [30]. From the fragmentation pattern, this compound was therefore identified as a propylated derivative of a monochlorinated dibenzofuran with one hydroxyl group.

### 3.3. Biotransformation of Chlorobenzyl Alcohols

**Figure 2:**
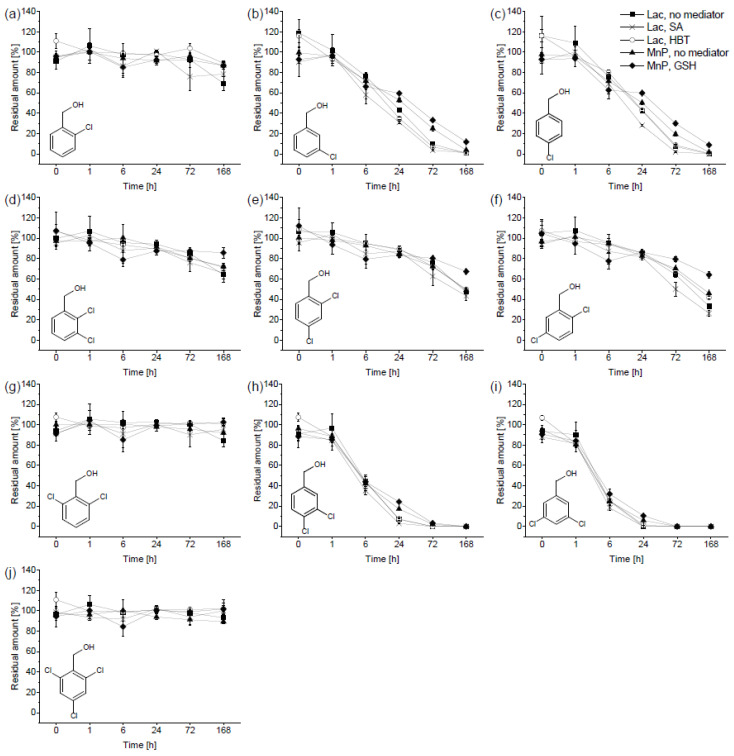
Residual amounts (related to corresponding heat-deactivated controls) of chlorobenzyl alcohols (CB-OHs) obtained during the in vitro experiment with the extracellular liquid of *Pleurotus ostreatus*: (**a**) 2-chlorobenzyl alcohol; (**b**) 3-chlorobenzyl alcohol; (**c**) 4-chlorobenzyl alcohol; (**d**) 2,3-dichlorobenzyl alcohol; (**e**) 2,4-dichlorobenzyl alcohol; (**f**) 2,5-dichlorobenzyl alcohol; (**g**) 2,6-dichlorobenzyl alcohol; (**h**) 3,4-dichlorobenzyl alcohol; (**i**) 3,5-dichlorobenzyl alcohol; and (**j**) 2,4,6-trichlorobenzyl alcohol. The CB-OHs were incubated in a mixture; the initial concentration was 2 µg mL^−1^ for each. The initial enzyme activities were 450 U L^−1^ of laccase and 30 U L^−1^ of manganese-dependent peroxidase (MnP). The laccase-favouring setup (Lac) contained no mediator (◼), syringaldehyde (SA; ✕), or 1-hydroxybenzotriazole (HBT; ◯); the MnP-favouring setup contained no mediator (▲) or glutathione (GSH; ⯁).

**Figure 3:**
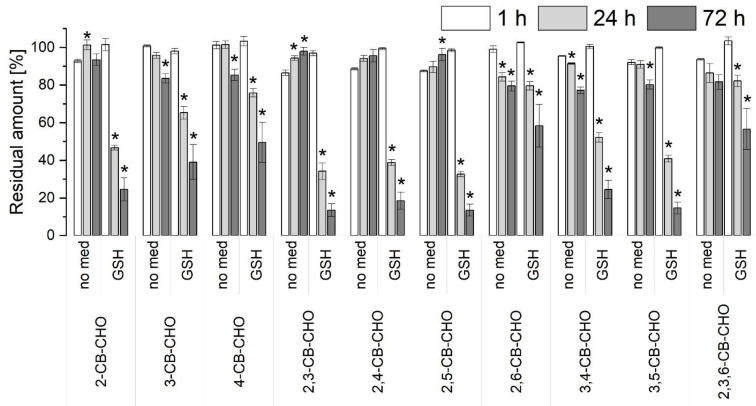
Residual amounts (related to corresponding heat-deactivated controls) of chlorobenzaldehydes (CB-CHOs) detected after incubation with the extracellular liquid of *Irpex lacteus* with no mediator (no med) or reduced glutathione (GSH): 2-chlorobenzaldehyde (2-CB-CHO); 3-chlorobenzaldehyde (3-CB-CHO); 4-chlorobenzaldehyde (4-CB-CHO); 2,3-dichlorobenzaldehyde (2,3-CB-CHO); 2,4-dichlorobenzaldehyde (2,4-CB-CHO); 2,5-dichlorobenzaldehyde (2,5-CB-CHO); 2,6-dichlorobenzaldehyde (2,6-CB-CHO); 3,4-dichlorobenzaldehyde (3,4-CB-CHO); 3,5-dichlorobenzaldehyde (3,5-CB-CHO); and 2,3,6-trichlorobenzaldehyde (2,3,6-CB-CHO). The CB-CHOs were incubated in a mixture; the initial concentration was 2 µg mL^−1^ for each. The initial enzyme activities were 706 U L^−1^ of manganese-dependent peroxidase and 114 U L^−1^ of manganese-independent peroxidase. The asterisks denote where significant (*p*-value < 0.05) differences were found between 1 h and 24 h or between 1 h and 72 h (ANOVA and Tukey’s range test).

**Figure 4:**
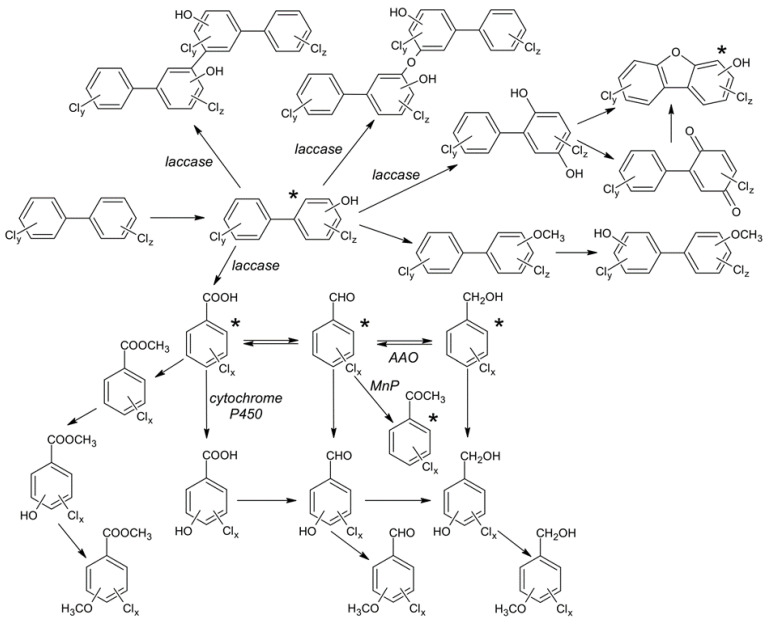
Pathways of the biotransformation of PCBs and their metabolites by ligninolytic fungi as proposed in the in vivo and in vitro studies by Čvančarová et al., Kamei et al., Muzikář et al., Schultz et al., Fujihiro et al., Kordon et al., Stella et al., and Ferreira et al. [1,2,4,18,19,20,21,31] and in this work. Where possible, enzyme involvement is stated. The asterisks denote compounds under study in the present work and the detected metabolites. MnP = manganese-dependent peroxidase; AAO = aryl-alcohol oxidase.

As shown in Figure 2, the extent of transformation by the extracellular liquid of *P. ostreatus* vastly varied among the CB-OHs. Unlike the biotransformation of OH-PCBs, the differences between different setups and mediated/nonmediated reaction mixtures were slight. The best removal was observed with 3,5-CB-OH (Figure 2i) and 3,4-CB-OH (Figure 2h). The transformation of 3-CB-OH (Figure 2b) and 4-CB-OH (Figure 2c)—albeit slower—was complete by the 7th day. Noticeably, the transformation of 2-CB-OH (Figure 2a) was minimal, suggesting that chlorine substituents in the *ortho*- position with respect to the functional group hinder the reaction. Indeed, incomplete transformation was found for 2,3-CB-OH (Figure 2d); 2,4-CB-OH (Figure 2e); and 2,5-CB-OH (Figure 2f). Moreover, no decrease in concentration was found for 2,6-CB-OH (Figure 2g) and 2,4,6-CB-OH (Figure 2j) with both *ortho*- positions bearing chlorine substituents.

For those CB-OHs that exhibited an apparent decrease in concentration (all except 2,6-CB-OH and 2,4,6-CB-OH; see Figure 2), corresponding CB-CHOs were detected (Figure S3). In accordance with the removal of CB-OHs, the differences in the formation of CB-CHOs between the setups were small; however, slightly lower amounts were found in the MnP-favouring setups than in the laccase-favouring setups. The fastest increase in concentration was observed for 3,4-CB-CHO (Figure S3g) and 3,5-CB-CHO (Figure S3h). Their maximal concentration (approximately equivalent to complete transformation of the CB-OHs) started to decrease after 24 h, which suggested further transformations of these isomers. In addition, the complete transformation of 3-CB-OH and 4-CB-OH after 7 days was confirmed by the detected amounts of 3-CB-CHO (Figure S3b) and 4-CB-CHO (Figure S3c), respectively.

### 3.4. Biotransformation of Chlorobenzaldehydes

In the primary in vitro experiment with CB-CHOs and the extracellular liquid of *P. ostreatus* containing mostly laccase, partial removal was found for some CB-CHOs only with the laccase setups, most noticeably for 3,4-CB-CHO and 3,5-CB-CHO (Figure S4). No differences between the mediated and nonmediated laccase-favouring reaction mixtures were found. No discernible decrease in concentration was found with the MnP-favouring setup.

To assess whether a higher activity of peroxidases can transform CB-CHOs to a further extent, an additional in vitro experiment was carried out with the extracellular liquid of *I. lacteus*, where the activity of MnP was approximately 20-fold higher than with *P. ostreatus*, but no laccase activity was detected. With no mediator, only a slight decrease in concentration was observed for some CB-CHOs after 72 h (3-CB-CHO; 4-CB-CHO; 2,6-CB-CHO; 3,4-CB-CHO; and 3,5-CB-CHO; Figure 3). However, MnP and/or MIP were able to transform all of the CB-CHO congeners with the addition of glutathione. The highest removals were observed for 2,3-CB-CHO; 2,4-CB-CHO; 2,5-CB-CHO; and 3,5-CB-CHO. In contrast to the transformation of CB-OHs with the extracellular enzymes of *P. ostreatus* (Figure 2), the suppressive effect of the chlorine substituents in the *ortho*- position (in relation to the functional group) could not be clearly distinguished with CB-CHOs.

In the nonderivatized samples after incubation of CB-CHOs with the extracellular liquid of *I. lacteus*, four peaks were detected with an *m*/*z* of 188 that exhibited an identical fragmentation pattern with initial losses of −15 (CH_3_) and −28 (CO) (Figure S5). These spectra were interpreted as different isomers of dichlorinated acetophenone. These compounds were found in trace amounts after 24 and 72 h of incubation, only in samples that contained active enzymes and added glutathione. Additionally, in the same set of samples, three peaks were found with similar fragmentation and an *m*/*z* of 154 belonging to monochlorinated acetophenones (Figure S6).

The biotransformation pathway of PCBs and their metabolites investigated in this and previous in vivo and in vitro studies is depicted in Figure 4. It is important to note that different fungal strains may employ/favour different pathways. Moreover, other intermediates are expected to be generated as a result of radical formation.

## 4. Discussion

### 4.1. Biotransformation of Hydroxylated Polychlorinated Biphenyls

There is evidence that some fungal laccases are able to transform PCBs with the addition of mediators such as ABTS [32]. Nevertheless, because of their phenolic nature, OH-PCBs are more suitable substrates for laccases [33]. However, as shown by the results (Figure 1), some OH-PCB congeners required the presence of mediators to be oxidized. Enzyme mediators are small compounds that facilitate electron transfer between an enzyme and a substrate that cannot be transformed by the enzyme itself, thereby substantially broadening its substrate range. In all instances, the addition of SA resulted in the fastest and most complete removal. As opposed to synthetic mediators, such as HBT or ABTS, SA is a compound widely present in the environment and can be obtained from lignin [34]. This may be of importance for applications of ligninolytic fungi or their enzymes in the remediation of PCBs and/or their transformation products. Nevertheless, the possible increase in toxicity caused by radicals generated from the oxidation of SA by laccase needs to be taken into account during potential bioremediation [35].

As expected, the removal of OH-PCBs was generally better for congeners with fewer chlorine substituents, which is in agreement with the literature [3,19]. As observed by Schultz et al. [18], the position of the hydroxyl group with respect to the chlorine substituents also plays a role in OH-PCB biotransformation and the formation of coupling products. In the present study, some differences were observed in the removal rates of 3-OH-2′,5′-PCB (Figure 1e) and 4-OH-2′,5′-PCB (Figure 1f), which differ only in the position of the hydroxyl group. In contrast, 2-OH-3,5-PCB (Figure 1d) exhibited identical behaviour to 4-OH-3,5-PCB (Figure 1g). Nevertheless, these isomers were nearly completely removed by the first sampling point (1 h), and any potential differences could have manifested at an earlier stage that was not monitored.

The rates of OH-PCB transformation are influenced by the structural molecular characteristics of individual congeners, which can be described with quantum chemical descriptors such as ionization energy (IE) or electronegativity [36]. This has already been demonstrated for OH-PCBs experimentally when a negative correlation was found between the IE of OH-PCBs and their removal rates by laccases of *P. ostreatus* and *T. versicolor* by Keum and Li [3]. The results of the removal of dichlorinated OH-PCBs that decreased in the order of 4-OH-3,5-PCB (IE = 8.812 eV) > 4-OH-2′,5′-PCB (IE = 8.872 eV) > 3-OH-2′,5′-PCB (IE = 8.902 eV) greatly correspond with this observation. Nevertheless, contrary to Keum and Li [3], 3-OH-2′,5′-PCB and 4-OH-2′,5′-PCB were not transformed at all without a mediator in the laccase setup. Additionally, the authors also suggested that chlorine atoms surrounding the hydroxyl group could cause steric hindrance and explain the lower rate constants of 4-OH-3,5-PCB that they observed; this is not supported by our results (Figure 1g). In fact, the removal of 4-OH-3,5-PCB without a mediator was better than that of some monochlorinated congeners (Figure 1b,c). One possible explanation is that laccase isoforms can differ in their substrate specificity and have higher affinities towards different OH-PCB isomers [19].

The transformation of OH-PCBs by the extracellular enzymes of *P. ostreatus* was substantially lower in the presence of reduced glutathione, which suggests that it acted as a laccase inhibitor. The activity of laccases produced by *Sinorhizobium meliloti* CE52G (Gram-negative bacterium) and the white-rot fungus *Daedalea quercina* have been previously found to be inhibited by reduced glutathione [37,38]. Glutathione was added with the intent of enhancing the reactions catalysed by MnP also present in the extracellular liquid. The fact that the same compound can serve as a mediator of one enzyme and an inhibitor of another also needs to be taken into consideration before any bioremediation applications. Noticeably, OH-PCBs were removed similarly or even better in the MnP-favouring setup with no mediator than in the laccase-favouring setup with no mediator. Combined, these results could signify either that laccase actually performed better in the MnP-favouring setup containing H_2_O_2_ and Mn^2+^ ions or that MnP was able to transform the OH-PCBs into intermediates more susceptible to subsequent laccase transformation. Moreover, MnP can generate radicals in the presence of malonate and Mn^2+^, which could have contributed to the removal of OH-PCBs [39,40,41].

During the biotransformation of OH-PCBs by laccase, dimer products, dechlorinated products, and quinones were detected as transformation products in previous studies [18,19,20]. Kamei et al. [2] also detected methoxylated PCBs from the in vivo degradation of OH-PCBs by *Phanerochaete* spp.; the production of metabolites was dependent on the particular strain as well as the type of cultivation medium (potato dextrose broth medium or low-nitrogen medium). In our case, only mono- to tri-chlorinated OH-PCBs were tested. Laccases have been shown to transform even higher chlorinated congeners, but this can only be associated with the formation of dimers [19]. Because the formation of dimeric products of OH-PCBs by laccase has already been well documented, we did not focus on these types of transformation products. During the degradation of OH-PCBs by the extracellular enzymes of *P. ostreatus*, a metabolite identified as a chlorinated hydroxydibenzofuran was found (Figure S2). A very similar mass spectrum was previously ascribed to 4-chloro-2-hydroxydibenzofuran, resulting from the degradation of 2-OH-3,5-CB by the laccase of *P. cinnabarinus* (Basidiomycota) but not by the laccase of *M. thermophila* (Ascomycota) [20]. In the present study, a mixture of 10 compounds was incubated with the extracellular enzymes; therefore, metabolite formation could not be assigned to a particular OH-PCB. The fact that this product could not be detected after 24 h of incubation suggests that it is transformed further and is not accumulated as a dead-end product. Moreover, apart from the dimerization of OH-PCBs, laccase is also probably able to degrade them into monoaromatic compounds, as evidenced by the identification of the monochlorinated CBA metabolite (Figure S1).

### 4.2. Biotransformation of Chlorobenzyl Alcohols

There is substantially less information about the transformation of CB-OHs and CB-CHOs, which have also been detected as intermediates of PCB degradation by ligninolytic fungi in a few studies [1,2,22]. In a previous study, evidence was found that MnP isoforms of *I. lacteus* can oxidize veratryl alcohol (3,4-dimethoxybenzyl alcohol) to veratraldehyde in the presence of Mn^2+^ ions and malonate or oxalate [39]. However, we did not observe any dependency of the transformation of CB-OHs on the presence of Mn^2+^ ions or H_2_O_2_, thereby excluding peroxidases in general. Moreover, only slight (if any) inhibition of the transformation by glutathione was observed for CB-OHs, and no improvement was found with the addition of laccase mediators, which also indicates that laccase was not responsible for the removal of CB-OHs. Therefore, because we used crude extracellular liquid, other extracellular enzymes of *P. ostreatus* that were not detected with the enzyme assays that we employed may have been involved. The suppressive effect of the chlorine substituents next to the functional group indicates that CB-OHs were direct substrates of the enzyme (as opposed to the reaction being mediated). In this respect, aryl-alcohol oxidases could be a possible explanation. These flavoproteins are produced by ligninolytic fungi, including *P. ostreatus*, and serve as one of the sources of H_2_O_2_ for peroxidases. Ferreira et al. [31] described a recombinant aryl-alcohol oxidase from *Pleurotus eryngii* that is capable of oxidizing 3-CB-OH and 4-CB-OH. Nevertheless, some aryl-alcohol oxidase assays detect the same conversion of veratryl alcohol to veratraldehyde [31,42] that was employed in this work to assess LiP, and no such activity was found.

### 4.3. Biotransformation of Chlorobenzaldehydes

Because of the accumulation of CB-CHOs that resulted from the transformation of CB-OHs, it was presumed that the predominant enzyme of *P. ostreatus*, laccase, is not able to oxidize these metabolites efficiently. Indeed, when CB-CHOs were used as substrates, a decrease in concentration was apparent only with 3,4-CB-CHO and 3,5-CB-CHO after 3 days of incubation in the laccase-favouring setups (Figure S4). Because the activity of MnP in the extracellular liquid of *P. ostreatus* was very low, to assess the true capability of peroxidases to transform CB-CHOs, an additional experiment was carried out with the extracellular liquid of *I. lacteus* containing MnP and MIP. Here, the removal was well improved by the addition of glutathione, which is a known MnP mediator that enables nonphenolic substrates to be oxidized by MnP, as has been shown, e.g., with the degradation of polycyclic aromatic hydrocarbons [27,43]. MnP is able to produce thiyl radicals from glutathione that participate in substrate conversions [44]. The fact that the hindering effect of the position of the chlorine substituents was not observed with CB-CHOs (compared to CB-OHs) is an additional indicator that the transformation occurred via the mediator.

## 5. Conclusions

It is expected that worldwide contamination by PCBs will over time result in their transformation into products, such as OH-PCBs, methoxylated PCBs, and other metabolites. Because these compounds can exhibit toxicity comparable to or higher than the corresponding PCBs, it is important to address this issue and gain information about further reactions of the daughter products. Ligninolytic fungi have many mechanisms by which they transform and degrade natural compounds as well as many recalcitrant pollutants. As has been shown with PCBs, the presence of the whole fungus with both extracellular and intracellular enzymatic apparatuses is needed to complete the degradation pathways. Nevertheless, in vitro experiments are invaluable for determining the responsible enzymes and assessing their potential and affinity for different isomers. A better description of the pathways can also help to avoid the accumulation of toxic metabolites during biodegradation applications. This work attempted to elucidate some of the missing steps in the biotransformation of metabolites of PCBs by ligninolytic enzymes. OH-PCBs, CB-OHs, and CB-CHOs were all transformed with certain limitations, such as the necessity for the presence of mediators or recalcitrance of some compounds. The transformation of various dichlorinated CB-OH and CB-CHO isomers is described here for the first time. In addition, this is also the first report of a monoaromatic compound (chlorobenzoic acid) detected in vitro as a metabolite in the degradation of OH-PCBs by ligninolytic enzymes.

## Data Availability

The data presented in this study are contained within the article and Supplementary Material.

## Supplementary Materials

The following are available online at https://www.mdpi.com/article/10.3390/toxics9040081/s1, Figure S1: Mass spectrum of a monochlorobenzoic acid detected after biotransformation of hydroxylated polychlorinated biphenyls by extracellular enzymes of *Pleurotus ostreatus*., Figure S2: Mass spectrum of a hydroxylated monochlorodibenzofuran detected after biotransformation of hydroxylated polychlorinated biphenyls by extracellular enzymes of *Pleurotus ostreatus*., Figure S3: Concentration of chlorobenzaldehydes detected during the biotransformation of chlorobenzyl alcohols (CB-OHs) by the extracellular liquid of *Pleurotus ostreatus.*, Figure S4: Residual amounts (related to corresponding heat-deactivated controls) of chlorobenzaldehydes (CB-CHOs) obtained during the biotransformation experiment with the extracellular liquid of *Pleurotus ostreatus.*, Figure S5: Mass spectrum of a dichlorinated acetophenone detected after biotransformation of chlorobenzaldehydes by extracellular enzymes of *Irpex lacteus*, and Figure S6: Mass spectrum of a monochlorinated acetophenone detected after biotransformation of chlorobenzaldehydes by extracellular enzymes of *Irpex lacteus*.
