# Supplementary material for: Degradation Products of Polychlorinated Biphenyls and Their In Vitro Transformation by Ligninolytic Fungi
Source: Toxics. 2021 Apr 8;9(4):81. doi: 10.3390/toxics9040081 (PMC8070434; doi:10.3390/toxics9040081)
Supplement: Supplementary file 1 [file toxics-09-00081-s001.pdf]

toxics

MDPI

*Article*

# Supplementary Material: Degradation Products of Polychlorinated Biphenyls and Their In Vitro Transformation by Ligninolytic Fungi

**Kamila Šrédlová, Kateřina Šírová, Tatiana Stella and Tomáš Cajthaml**

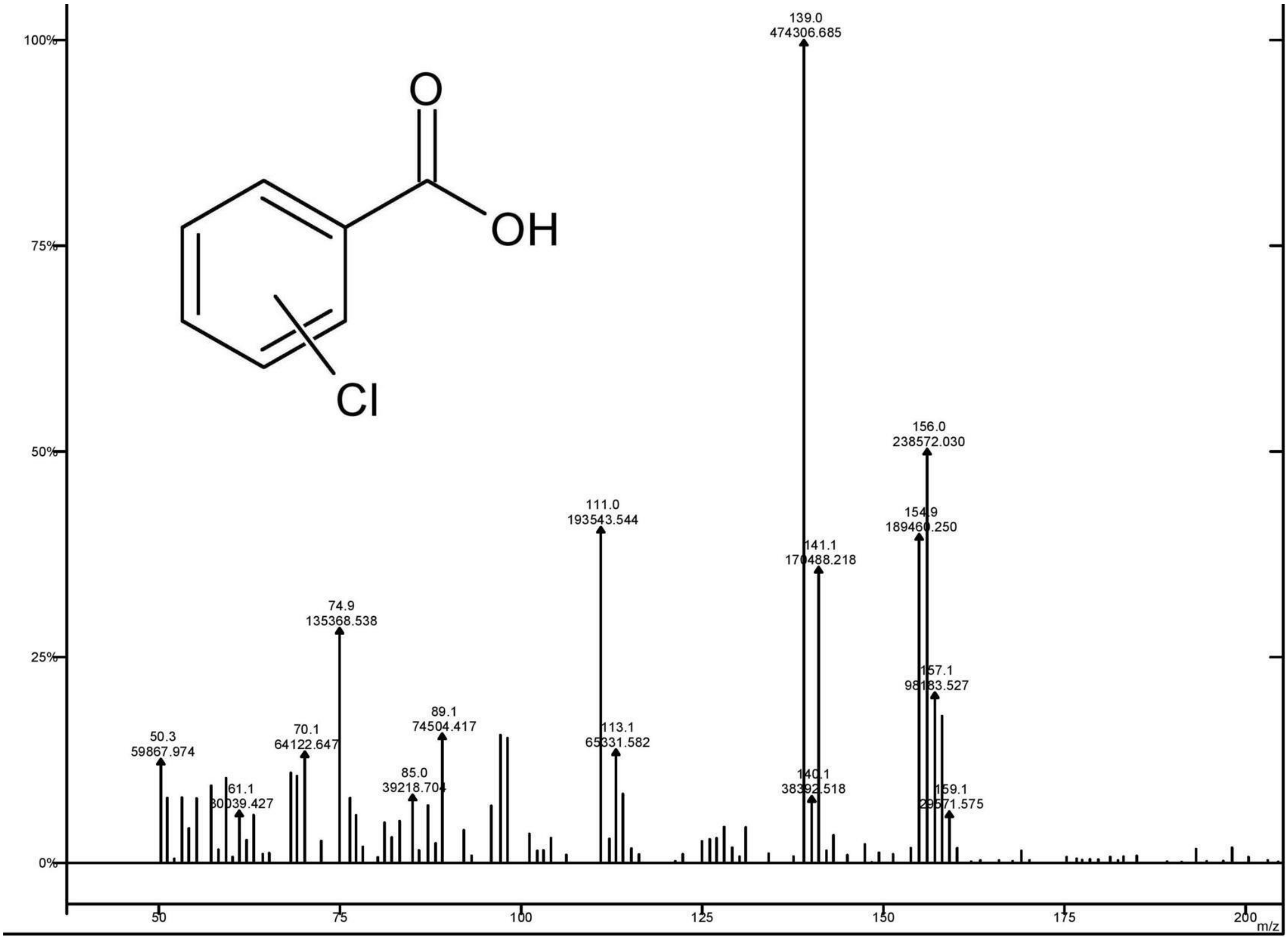


**Figure S1.** Mass spectrum of a monochlorobenzoic acid detected after biotransformation of hydroxylated polychlorinated biphenyls by extracellular enzymes of *Pleurotus ostreatus*.

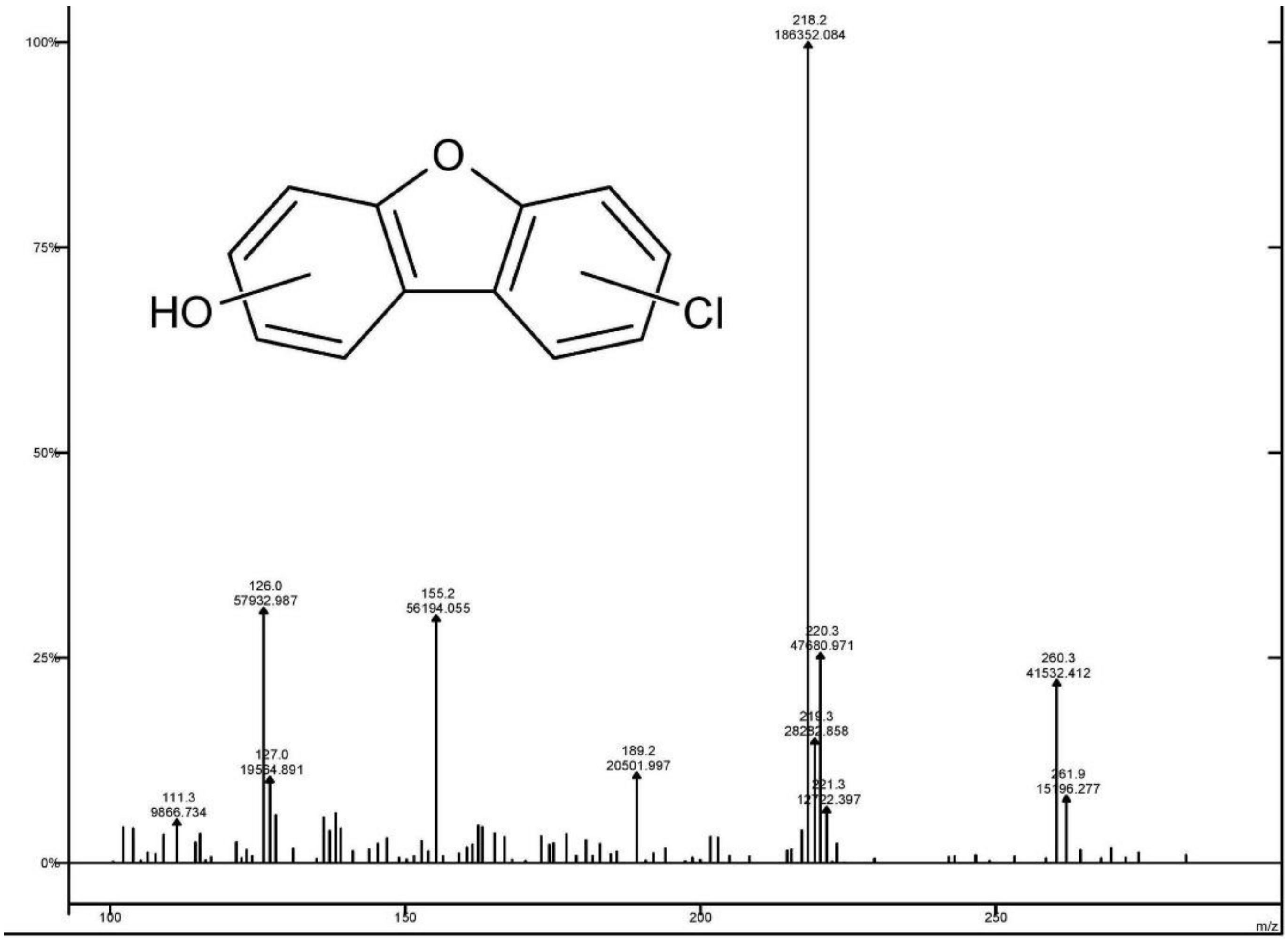


**Figure S2.** Mass spectrum of a hydroxylated monochlorodibenzofuran detected after biotransformation of hydroxylated polychlorinated biphenyls by extracellular enzymes of *Pleurotus ostreatus*.

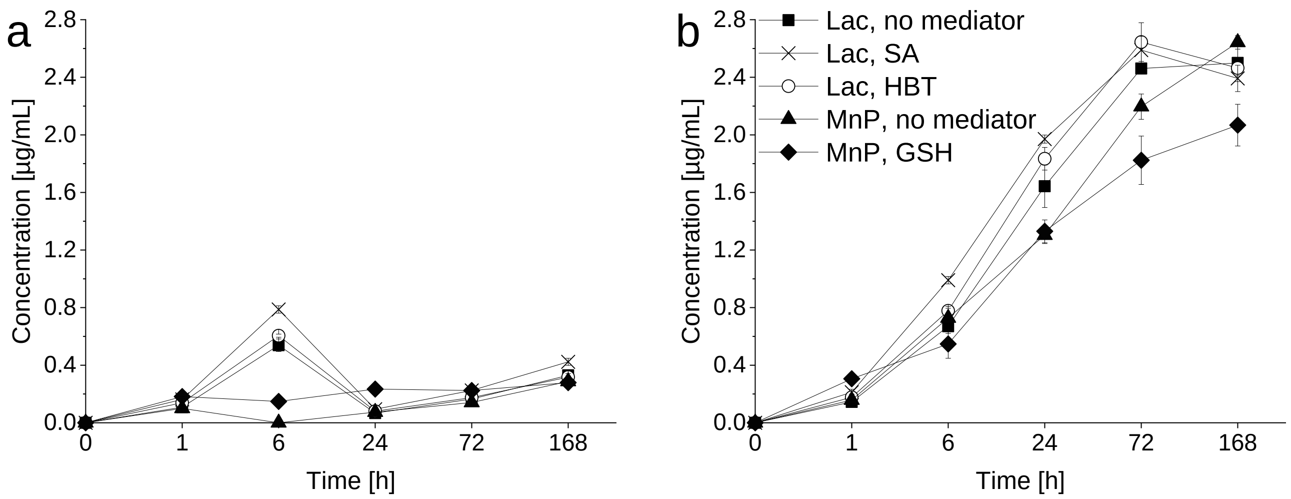

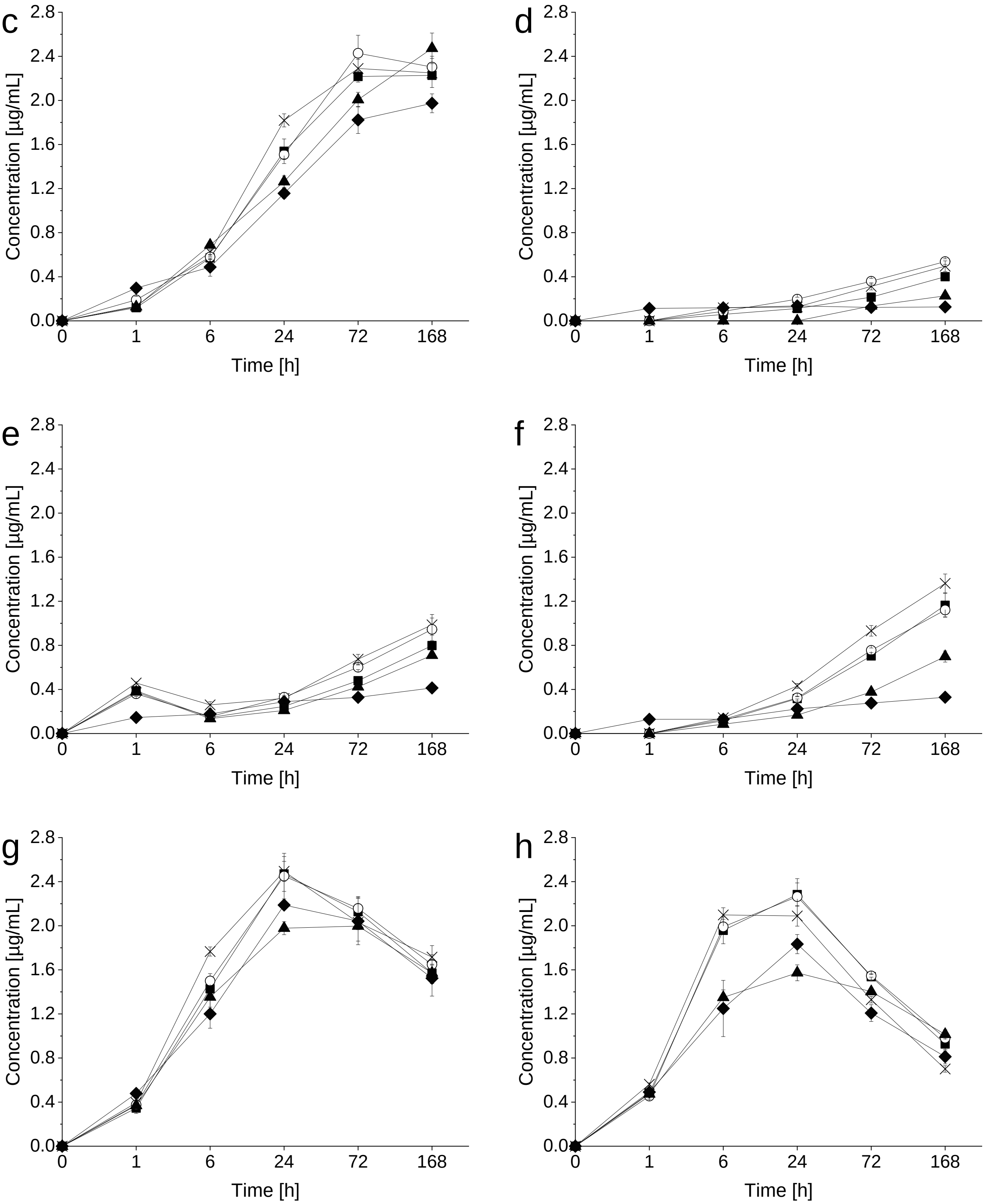


**Figure S3.** Concentration of chlorobenzaldehydes detected during the biotransformation of chlorobenzyl alcohols (CB-OHs) by the extracellular liquid of *Pleurotus ostreatus*: 2-chlorobenzaldehyde (**a**); 3-chlorobenzaldehyde (**b**); 4-chlorobenzaldehyde (**c**); 2,3-dichlorobenzaldehyde (**d**); 2,4-dichlorobenzaldehyde (**e**); 2,5-dichlorobenzaldehyde (**f**); 3,4-dichlorobenzaldehyde (**g**); and 3,5-dichlorobenzaldehyde (**h**). The CB-OHs were degraded in a mixture; initial concentration was 2 μg $mL^{-1}$ of each. Initial enzyme activity was 450 U $L^{-1}$ of laccase and 30 U $L^{-1}$ of manganese-dependent peroxidase (MnP). The laccase-favouring setup (Lac) contained no mediator (■), syringaldehyde (SA; ╳), or 1-hydroxybenzotriazole (HBT; ◯); the MnP-favouring setup contained no mediator (▲) or glutathione (GSH; ◆).

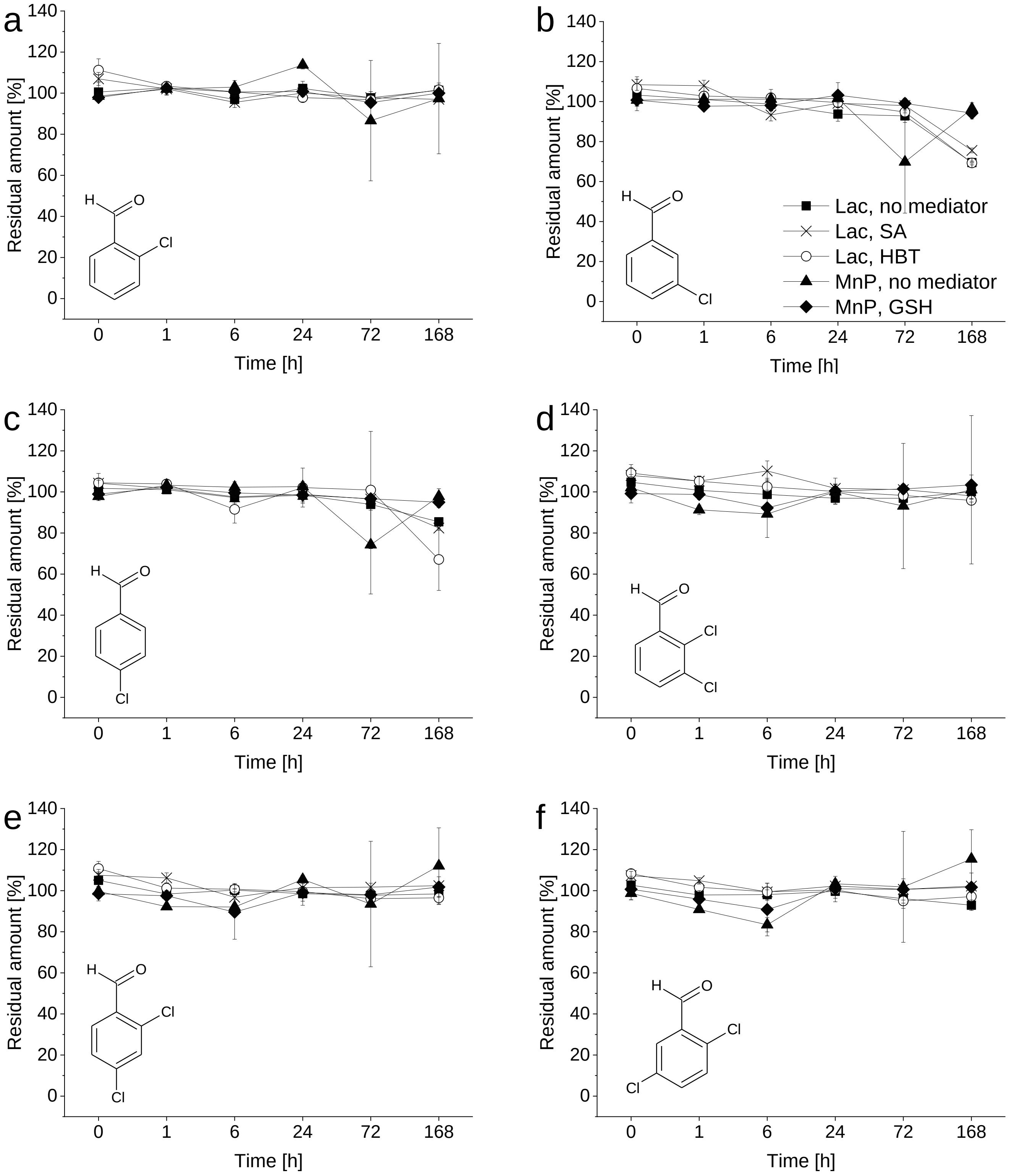
a
b
c
d
e
f
Residual amount [%]
Time [h]
0
1
6
24
72
168
140
120
100
80
60
40
20
0
H
O
Cl
Lac, no mediator
Lac, SA
Lac, HBT
MnP, no mediator
MnP, GSH

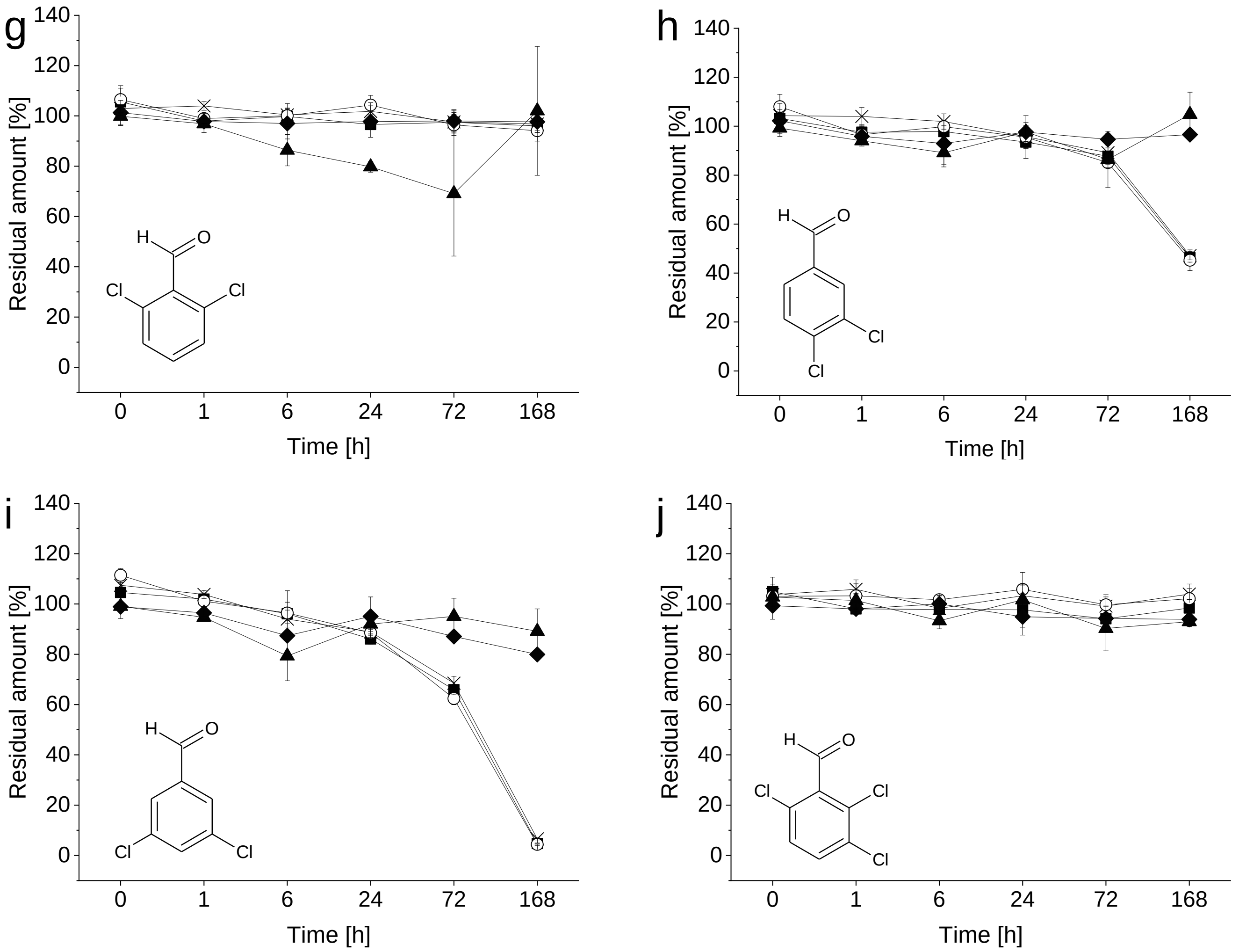


**Figure S4.** Residual amounts (related to corresponding heat-deactivated controls) of chlorobenzaldehydes (CB-CHOs) obtained during the biotransformation experiment with the extracellular liquid of *Pleurotus ostreatus*: 2-chlorobenzaldehyde (**a**); 3-chlorobenzaldehyde (**b**); 4-chlorobenzaldehyde (**c**); 2,3-dichlorobenzaldehyde (**d**); 2,4-dichlorobenzaldehyde (**e**); 2,5-dichlorobenzaldehyde (**f**); 2,6-dichlorobenzaldehyde (**g**); 3,4-dichlorobenzaldehyde (**h**); 3,5-dichlorobenzaldehyde (**i**); and 2,3,6-trichlorobenzaldehyde (**j**). The CB-CHOs were degraded in a mixture; initial concentration was 2 μg $mL^{-1}$ of each. Initial enzyme activity was 450 U $L^{-1}$ of laccase and 30 U $L^{-1}$ of manganese-dependent peroxidase (MnP). The laccase-favouring setup (Lac) contained no mediator (■), syringaldehyde (SA; ╳), or 1-hydroxybenzotriazole (HBT; ◯); the MnP-favouring setup contained no mediator (▲) or glutathione (GSH; ◆).

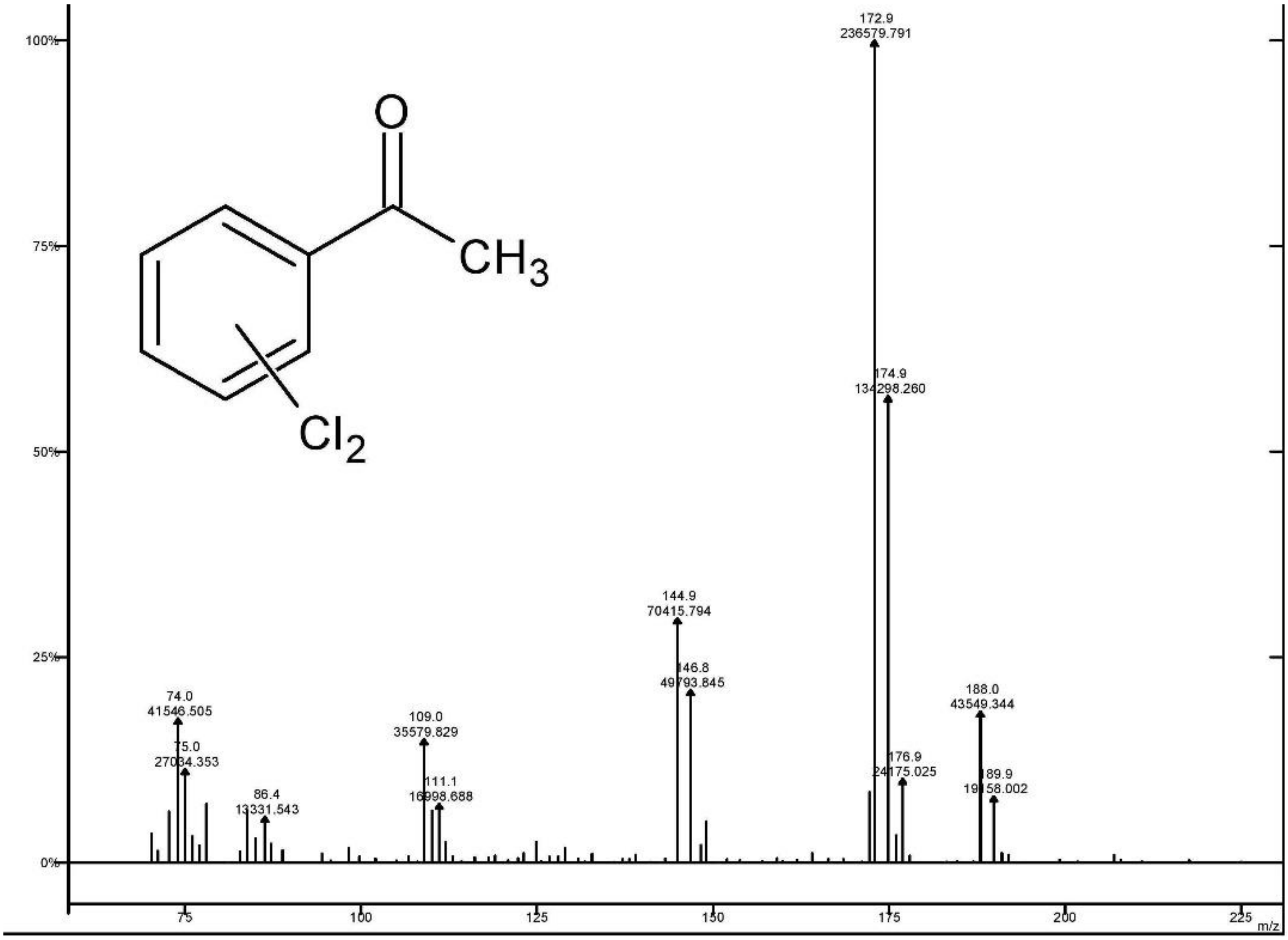


**Figure S5.** Mass spectrum of a dichlorinated acetophenone detected after biotransformation of chlorobenzaldehydes by extracellular enzymes of *Irpex lacteus*.

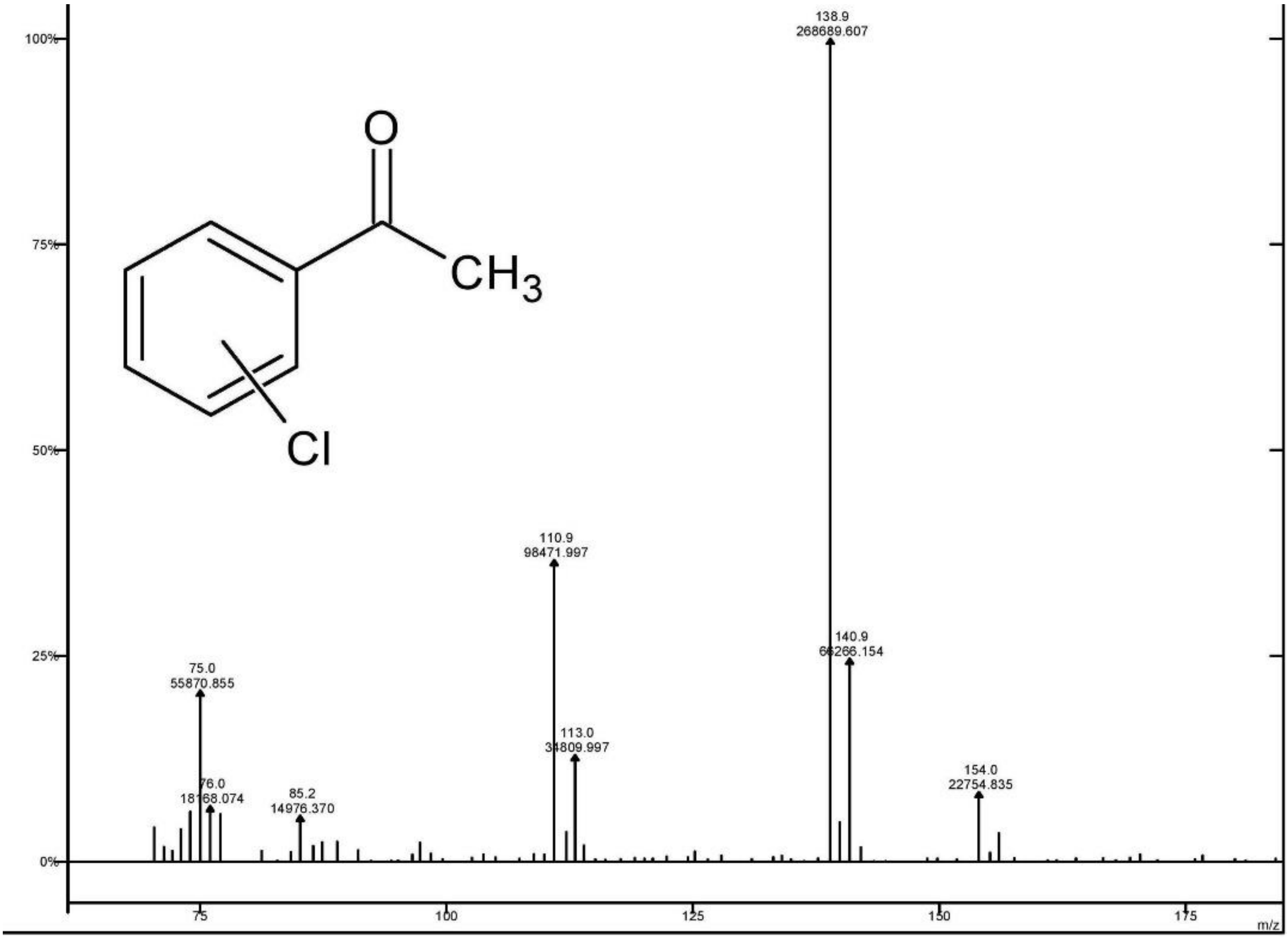


**Figure S6.** Mass spectrum of a monochlorinated acetophenone detected after biotransformation of chlorobenzaldehydes by extracellular enzymes of *Irpex lacteus*.
